# Neurocognitive effects of six ketamine infusions and the association with antidepressant effects in treatment-resistant bipolar depression: a preliminary study

**DOI:** 10.7717/peerj.10208

**Published:** 2020-11-03

**Authors:** Wei Zheng, Yan-Ling Zhou, Cheng-Yu Wang, Xiao-Feng Lan, Bin Zhang, Ming-Zhe Yang, Sha Nie, Yu-Ping Ning

**Affiliations:** 1 The Affiliated Brain Hospital of Guangzhou Medical University, Guangzhou, China; 2 The First School of Clinical Medicine, Southern Medical University, Guangzhou, Guangdong, China, Guangzhou, China

**Keywords:** Ketamine, MCCB, Neurocognition, Bipolar depression

## Abstract

**Objective:**

The N-methyl-D-aspartate subtype glutamate receptor antagonist ketamine has rapid antidepressant and antisuicidal effects in treating treatment-resistant bipolar depression (TRBD). The neurocognitive effects of repeated ketamine infusions in TRBD are not known.

**Methods:**

Six intravenous infusions of ketamine (0.5 mg/kg over 40 min) were administered on a Monday–Wednesday–Friday schedule during a 12-day period on 16 patients with TRBD followed by a 2-week observational period. The assessment of neurocognitive function was conducted using the MATRICS Consensus Cognitive Battery at baseline, 13 and 26 days. Tasks were designed to test speed of processing, working memory, visual learning and verbal learning.

**Results:**

A significant improvement was found only in scores of speed of processing (*F* = 9.9, *p* = 0.001) after a 2-week observational period, which was accounted for by the improvement of depression symptoms. There were no significant changes over time in terms of working memory, visual learning and verbal learning. Pearson correlation analysis showed that the improvement of depression symptoms through six ketamine infusions was greater among TRBD patients with lower working memory at baseline (*r* = 0.54, *p* = 0.03). In multiple regression analysis, the significant correlation was still maintained (beta = 0.67, *t* = 2.2, *p* = 0.04).

**Conclusion:**

This preliminary study indicated that six ketamine infusions were not harmful but were slightly beneficial for speed of processing in TRBD. However, this change was mainly accounted for the improvement of depression symptoms over time. Lower baseline working memory appears to be associated with greater antidepressant response after completion of six ketamine infusions in patients with TRBD.

## Introduction

Bipolar disorder (BD) is one of the leading causes of disability worldwide, with a prevalence ranging from 1% to 2% in the adult population (Weissman et al., 1988). Traditional mood stabilizers (such as valproate, lithium, and carbamazepine) and antipsychotics (such as olanzapine) are effective in treating acute manic episodes and preventing relapse or recurrence (Cipriani et al., 2013; Miura et al., 2014). However, pharmacological treatment options for bipolar depression are weak, particularly for treatment-resistant bipolar depression (TRBD) (Thase, 2006). In recent decades, ketamine, a noncompetitive antagonist of the N-methyl-D-aspartate (NMDA) subtype glutamate receptor, has showed rapid and robust antisuicidal and antidepressant effects in TRBD (Diazgranados et al., 2010; Grunebaum et al., 2017; McCloud et al., 2015; Rybakowski, Permoda-Osip & Bartkowska-Sniatkowska, 2017). Growing evidence has consistently reported that repeated ketamine infusions had achieved greater rates of response and remission compared to a single ketamine infusion (Murrough et al., 2013; Rasmussen et al., 2013; Shiroma et al., 2014b; Zheng et al., 2020), but the neurocognitive effects of repeated ketamine infusions have raised some concerns.

In two early studies (Krystal et al., 1994; Parwani et al., 2005) focusing on healthy volunteers, ketamine administration at subanaesthetic doses was associated with neurocognitive impairments, particularly for memory and learning. Selective impairments in aspects of executive functioning had been reported in prior studies (Krystal et al., 1994), but not in all studies (Morgan et al., 2004; Parwani et al., 2005). Ketamine at subanaesthetic doses seems to carry a very low risk of neurocognitive deficits in healthy subjects (Perry et al., 2007), supported by the lack of long-term neurocognitive effects in studies with anaesthetic doses of ketamine (Moretti et al., 1984).

The neurocognitive effects of repeated infusions of ketamine have just started to be examined in major depressive disorder (MDD) or mood disorders, but the findings were inconsistent. For example, Shiroma et al. (2014a) reported improvements in visual memory and simple and complex working memory after 15 subjects with treatment-resistant major depression (TRD) completed six ketamine infusions. Another study found no significant memory deficits in patients with TRD (78.6%, 22/28) and TRBD (21.4%, 6/28) treated with three or six ketamine infusions over 3 weeks (Diamond et al., 2014). However, the short-term neurocognitive effects of repeated intravenous infusions of ketamine at subanaesthetic doses targeting on TRBD are not known. Importantly, Zhou et al. (2018) found that greater baseline visual learning could predict the antidepressant effects of six ketamine infusions for patients with unipolar and bipolar depression.

Therefore, we aimed to examine the neurocognitive effects of six intravenous infusions of ketamine and whether neurocognitive performance at baseline were associated with the change in depression severity in TRBD. We hypothesized that six ketamine infusions at subanaesthetic doses were devoid of neurocognitive impairments and that baseline neurocognitive function would be the predictor of ketamine response in TRBD.

## Methods

This neurocognitive study was conducted during a single-arm open-label study of repeated intravenous infusions of ketamine in TRBD (ChiCTR-OOC-17012239). This study was conducted in accordance with the Declaration of Helsinki. The study protocol was approved by the Ethics Committee of the Affiliated Brain Hospital of Guangzhou Medical University (Ethical Application Ref: 2016-030). Written informed consent was obtained from all subjects.

### Participants

All subjects were recruited from the Affiliated Brain Hospital of Guangzhou Medical University, a psychiatric hospital owned by Guangzhou city, between September 2016 and December 2017. The following inclusion criteria of this single-arm open-label study included (1) both genders; (2) aged 18–65 years, Han Chinese; (3) diagnosis of non-psychotic BD was assessed by two experienced psychiatrists at study entry, which was subsequently confirmed by using a checklist following the Diagnostic and Statistical Manual of Mental Disorders, fifth edition criteria; (4) Hamilton Depression Rating Scale-17 items (HAMD-17) total score ≥17 (Hamilton, 1960; Xie & Shen, 1984) at screening; and (5) lack of response to ≥2 adequate antidepressant treatment trials during the current episode of depression (Diamond et al., 2014).

The following exclusion criteria of this single-arm open-label study included (1) lifetime history of alcohol dependence or substance abuse and other serious mental disorders such as psychotic illness; however, this study allowed a comorbidity of anxiety, panic or phobia obsessive-compulsive disorders if it was not the primary presenting problem in the previous 1 year; (2) unstable medical or neurological illness; (3) serious and imminent suicidal or homicidal risk; (4) pregnant or breast feeding; and (5) positive urine toxicology.

### Study design

Subjects with TRBD received six intravenous infusions of 0.5 mg/kg ketamine over 40 min using a single-arm open-label study design following the treatment regimen reported in Shiroma et al. (2014b) study. The detailed description of the methodology of this study has been published previously (Zheng et al., 2019). For all patients, their psychotropic medications were prescribed at the stable dosage for ≥4 weeks before conducting this study, and they continued to take the same treatment regimen during the whole research process. Nonpharmacological interventions, including repeated transcranial magnetic stimulation, electroconvulsive therapy and other psychotherapy methods, were not allowed in this study.

### Depression symptoms assessment

Change in depression severity was assessed by using the 10-item Montgomery-Asberg Depression Rating Scale (MADRS) at days 0, 13 (the last infusion) and 26 (a 2-week follow-up after TRBD patients completed the last ketamine infusion) (Montgomery & Asberg, 1979; Zhong et al., 2011). The clinical raters received concurrent training on the MADRS before the start of the study. After training, repeated assessments achieved an interrater correlation coefficient of at least 0.9 for the MADRS.

### Neurocognitive assessment

We applied the MATRICS Consensus Cognitive Battery (MCCB) to each subject, measuring neurocognitive function at baseline, 13 and 26 days (Green & Nuechterlein, 2004; Nuechterlein et al., 2008). Given that neurocognitive dysfunctions in domains of verbal learning and memory tasks are prominent in BD (Bourne et al., 2015), four domains of the MCCB (including verbal learning, visual learning, working memory, and speed of processing) were utilized in this study. We created a standardized score for the four domains of the MCCB data (*T* scores with a mean of 50 and a standard deviation (SD) of 10). The United States Food and Drug Administration (FDA) has recommended the MCCB as the primary assessment instrument of neurocognitive performance for schizophrenia (Nuechterlein et al., 2008), and a recent meta-analysis including seven case-control studies (*n* = 1,057) showed that the MCCB can also be usefully applied to assess neurocognitive dysfunction for BD (Bo et al., 2017).

### Statistical analysis

Following the methodology of a previous study (Zheng et al., 2019), only subjects who completed six ketamine intravenous infusions and the assessment of neurocognitive performance at days 0 and 13 were included in the final analyses, which were performed using SPSS 24.0. Linear mixed models were applied to examine the change in MADRS score and four domains of neurocognitive performance over time. Linear mixed models were also repeated by controlling for MADRS change score over time to analyse whether changes in neurocognitive performance following six ketamine infusions were attributed to the improvement of depression symptoms. Correlations of neurocognitive function at baseline and changes in MADRS score following six ketamine infusions were examined by using Pearson correlation coefficients. After then, a stepwise multiple regression analysis was conducted to investigate the degrees of correlation after controlling for the following independent variables, including the demographic and clinical variables, such as duration of illness, age of onset, age, and family history of psychiatric disorders following the methodology of the earlier study (Shiroma et al., 2014a). To adjust for multiple testing, Bonferroni corrections were also used in this study (e.g., *m* = 1 × 4 = 4, α = 0.05/4 = 0.0125). Significance levels were set at 0.05 for all statistical analyses.

## Results

Altogether, 19 patients with TRBD were enrolled and received repeated ketamine infusions in this study. Of them, three patients dropped out after the first infusion due to inefficacy (*n* = 1), after two infusions due to the withdrawal of consent (*n* = 1), and after the fourth infusion due to inefficacy (*n* = 1). All statistical analyses in this study were limited to those 16 subjects with TRBD who completed six ketamine infusions and neurocognitive tasks measured at days 0 and 13.

### Demographic and clinical characteristics

The demographic and clinical characteristics of each sample are presented in Table S1. The sample age ranged from 19 to 62 years, age of onset ranged from 13 to 40 years, and illness duration ranged from 37 to 408 months. Five had at least one past history of psychiatric hospitalization, and 10 had a family history of psychiatric disorders. The MADRS mean scores ranged from 15 to 44.

### Antidepressant outcomes after six ketamine infusions

In linear mixed models summarized in Table 1, the changes in MADRS scores over time are shown, with a significant main effect of time (*F* = 51.8, *p* < 0.001), where depression severity significantly decreased from baseline (mean MADRS score = 28.2) to day 13 (mean MADRS score = 8.4, *p* < 0.001) and to day 26 (mean MADRS score = 11.2, *p* < 0.001).

**Table 1:** Depressive symptoms and neurocognitive performance at days 0, 13, and 26.

Outcomes	Day 0		Day 13		Day 26		Time *F* (*p*)	Day 13 vs. day 0		Day 26 vs. day 0	
	Mean	SD	Mean	SD	Mean	SD		*p*	Cohen’s d	*p*	Cohen’s d
MADRS score	28.2	8.3	8.4	6.2	11.2	6.4	51.8 (**<0.001**)	**<0.001**	**2.7**	**<0.001**	**2.3**
Speed of processing	40.4	14.1	48.1	15.0	48.8	12.0	9.9 (**0.001**)^a^	**0.007**	**−0.5**	**0.002**	**−0.6**
Working memory	44.3	13.0	45.9	11.4	44.6	12.0	0.2 (0.82)	1.00	−0.1	1.00	−0.02
Verbal learning	39.8	15.0	45.9	12.4	39.5	10.5	2.9 (0.08)	0.12	−0.4	1.00	0.02
Visual learning	43.1	13.2	42.2	9.1	44.0	8.4	0.2 (0.85)	1.00	0.08	1.00	−0.08

**Note:**

a Significant change over time disappeared after repeating the linear mixed model analysis of speed of processing (*F* = 1.1, *p* = 0.36) with MADRS change score as a covariate. Bolded values are *p* < 0.05. Abbreviations: MADRS, Montgomery-Asberg Depression Rating Scale.

### Neurocognitive effects after six ketamine infusions

In linear mixed models summarized in Table 1 and Fig. S1, the speed of processing over time is shown, with a significant main effect of time (*F* = 9.9, *p* = 0.001), where the score in speed of processing significantly increased from baseline (mean score in speed of processing = 40.4) to day 13 (mean score in speed of processing = 48.1, effect size = 0.5, *p* = 0.007) and to day 26 (mean score in speed of processing = 48.8, effect size = 0.6, *p* = 0.002). Other neurocognitive tasks showed no significant changes over time. When the linear mixed models were re-analysed using MADRS change score as a covariate, the significant findings disappeared (*F* = 1.1, *p* = 0.36).

### Baseline cognitive function predicts depression severity after six ketamine infusions

**Figure 1:**
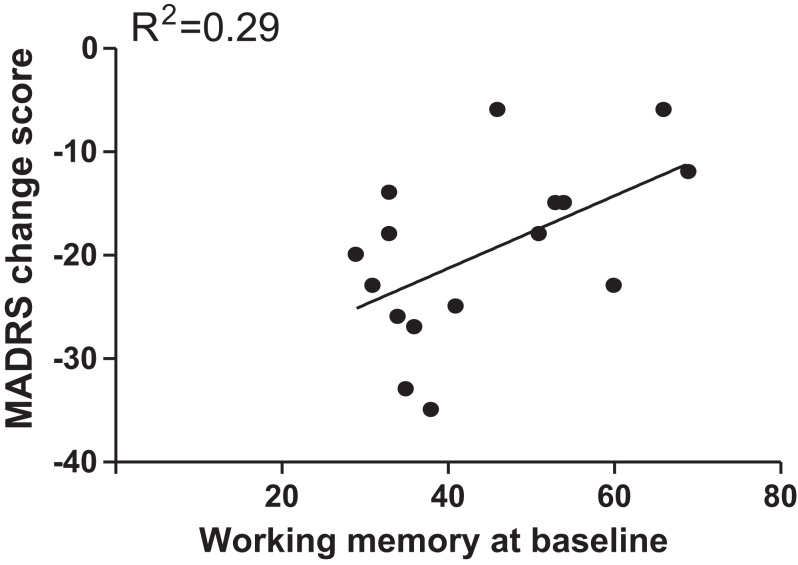
Correlation analyses to examine the association of MADRS change scores with baseline scores of working memory.

Pearson correlation analysis showed that only working memory score at baseline (*r* = 0.54, *p* = 0.03) was a significant predictor for change in depression severity, but it did not pass Bonferroni correction (*m* = 1 × 4 = 4, α = 0.05/4 = 0.0125) (Fig. 1). However, the significant correlation was still maintained (beta = 0.67, *t* = 2.2, *p* = 0.04) after controlling for duration of illness, age of onset, age, and family history of psychiatric disorders. As shown in Fig. S2, we did not find any significant correlations between MADRS change score and other baseline cognitive functions.

## Discussion

To the best of our knowledge, this is the first study to investigate the neurocognitive effects of six ketamine infusions and their association with change in depression severity in TRBD. Our findings suggested that no neurocognitive deficits were found when patients with TRBD received six infusions of ketamine at subanaesthetic doses. In contrast, repeated ketamine infusions were significantly related to the improvement of speed of processing measured by using the MCCB; this effect was attributed to the improvement of depression severity over time. Importantly, the improvement of depression severity was positively associated with lower working memory at baseline.

Growing evidence has demonstrated that repeated-dose infusions of ketamine can produce neurocognitive impairments and increase impulsive behaviours in animal studies (Ding et al., 2016; Melo et al., 2016). However, a growing number of clinical studies have consistently found that repeated ketamine infusions at subanaesthetic doses had a rapid-onset and sustained antidepressant effects (Murrough et al., 2013; Rasmussen et al., 2013; Shiroma et al., 2014b). Shiroma et al. (2014b) reported that up to 91.6% and 66.7% of patients with TRD achieved the response and remission criteria following the addition of five ketamine infusions, although residual neurocognitive deficits were always noted in remission states of mood disorders (Bas et al., 2015; Bortolato, Carvalho & McIntyre, 2014; Roux et al., 2017). Thus, this study was designed to investigate whether the antidepressant effects of ketamine may improve neurocognitive deficits in TRBD. In this study, the improvement of speed of processing was found after six ketamine infusions were completed, but this change was attributed to the improvement of depression severity over time. Residual depression symptoms and neurocognitive deficits were the most prominent risk factors and were negatively associated with psychosocial functioning among patients with BD (Bas et al., 2015). However, our findings may potentially indicate that neurocognitive impairments were often the last and residual symptoms, even when residual depression symptoms had been significantly improved. As shown in Table 1, there was an increase in verbal learning score, but it decreased thereafter; the reasons for the current findings are as yet unclear.

Numerous studies have indicated that the neurocognitive effects of ketamine were either beneficial or harmful, which can be partially attributed to the dose of ketamine (Browne & Lucki, 2013; Li et al., 2010). In animal studies, the subanaesthetic dose (10 mg/kg) of a single ketamine infusion, but not the anaesthetic dose (80 mg/kg), could rapidly activate the mammalian target of rapamycin (mTOR) signalling pathway and increase brain-derived neurotrophic factor (BDNF) levels, which could be related to its rapid antidepressant effects in TRBD (Autry et al., 2011; Li et al., 2010). Growing evidence has consistently found that both hippocampal synaptic plasticity and BDNF are involved in neurocognitive performance (Kim et al., 2017; Lu, Nagappan & Lu, 2014). Conversely, the anaesthetic dose of ketamine infusion (80 mg/kg), but not the lower dose (30 mg/kg), could increase the number of apoptotic cells and the miR-214 levels and impair memory and learning (Wang et al., 2014). Furthermore, neurocognitive impairments were found by the cumulative dose of ketamine. For example, subanaesthetic ketamine following a 6-month administration paradigm significantly resulted in spatial reference memory deficits (Ding et al., 2016). Importantly, chronic ketamine abusers were associated with decreased hippocampal function (Morgan et al., 2014) and BDNF (Ke et al., 2018). Taken together, six ketamine infusions at subanaesthetic doses over a 12-day period were not associated with neurocognitive impairments, which was supported by the prior study (Shiroma et al., 2014a).

In this study, baseline neurocognitive function was significantly associated with the improvement of depression symptoms in patients with TRBD who completed six ketamine infusions, as supported by the earlier study (Shiroma et al., 2014a). Shiroma et al. (2014a) reported that the likelihood of response to repeated-dose infusions of ketamine was significantly greater in depressed patients with baseline lower attention. However, Diamond et al. reported that ketamine did not cause memory impairments, as measured the Autobiographical Memory Interview—Short Form (AMI-SF) and the Autobiographical Fluency Task (AFT) when administered on up to six occasions (Diamond et al., 2014). This discrepancy could be partially accounted for by the assessment instruments of neurocognitive function (the AMI-SF and AFT were utilized in the study by Diamond et al. (2014) compared with the MCCB utilized in this study).

Several limitations in this study should be acknowledged. First, in this single-arm, open-label study, the lack of a control group was a limitation because it is helpful to exclude the effect of natural changes in patients’ neurocognitive function. Second, the subject numbers were relatively small in this study. Third, the current study was an add-on ketamine (0.5 mg/kg over 40 min) study because patients with TRBD continued to take their prior prescribed medications throughout the whole study, which may have enhanced or masked the effect of ketamine. However, their psychotropic medication dosages were stable for more than 1 month before starting the research. Thus, neurocognitive effects of ketamine can be explained appropriately by the add-on effect of repeated ketamine infusions for TRBD. Fourth, neurocognitive function was assessed using the MCCB in this study, but the optimal biomarker-based assessments were possibly attributed to multi-modal data acquisition, such as quantitative electroencephalography (Cook et al., 2013), peripheral blood markers (Uddin, 2014), and neuroimaging (Kambeitz et al., 2017). Furthermore, the P300 is correlated with working memory, use of neurocognitive resources, and target recognition (Fu et al., 2018). Despite unobservable cognitive impairments, several studies reported that it can be found by the P300 event-related potential component (Ozen et al., 2013). Thus, the P300 is better than a traditional cognitive test that relies on the subjective judgement of trained professionals. Fifth, Hu et al. (2016) reported that the Young Mania Rating Scale (YMRS) scores increased significantly with ketamine augmentation (1 and 2 h). However, data of mania-like symptoms were not collected in this study.

In conclusion, this preliminary study indicated that six ketamine infusions at subanaesthetic doses did not impair the neurocognitive performance in TRBD. Importantly, lower working memory at baseline appears to be positively associated with the likelihood of antidepressant response following six ketamine infusions in TRBD.

## Supplemental Information

10.7717/peerj.10208/supp-1 Supplemental Information 1 Changes in cognitive performance scores in patients with treatment-resistant bipolar disorder.

10.7717/peerj.10208/supp-2 Supplemental Information 2 Demographic and clinical characteristics of patients with treatment-resistant bipolar disorder.

10.7717/peerj.10208/supp-3 Supplemental Information 3 Correlation analyses to examine the association of MADRS change score with baseline scores of speed of processing, visual learning, and verbal learning.

10.7717/peerj.10208/supp-4 Supplemental Information 4 Raw Data with a codebook.

## Additional Information and Declarations

### Clinical Trial Registration

The following information was supplied regarding Clinical Trial registration: ChiCTR-OOC-17012239.

### Data Availability

The following information was supplied regarding data availability: Raw data are available as a Supplemental File.
