# Supplementary figures and images for: Neurocognitive effects of six ketamine infusions and the association with antidepressant effects in treatment-resistant bipolar depression: a preliminary study
Source: PeerJ. 2020 Nov 3;8:e10208. doi: 10.7717/peerj.10208 (PMC7646297; doi:10.7717/peerj.10208)

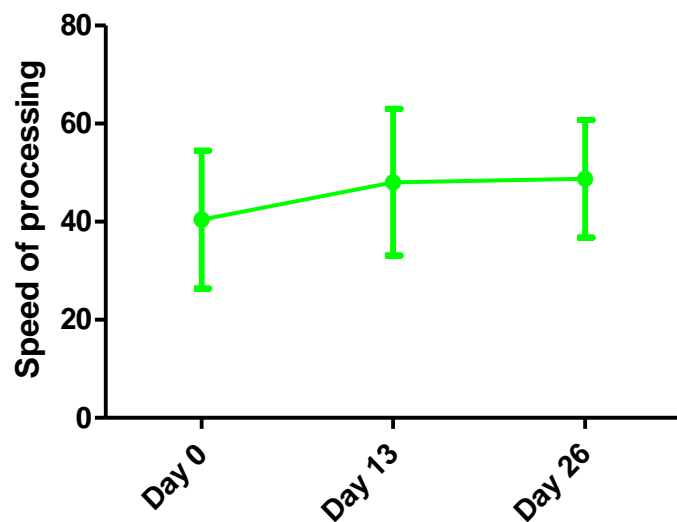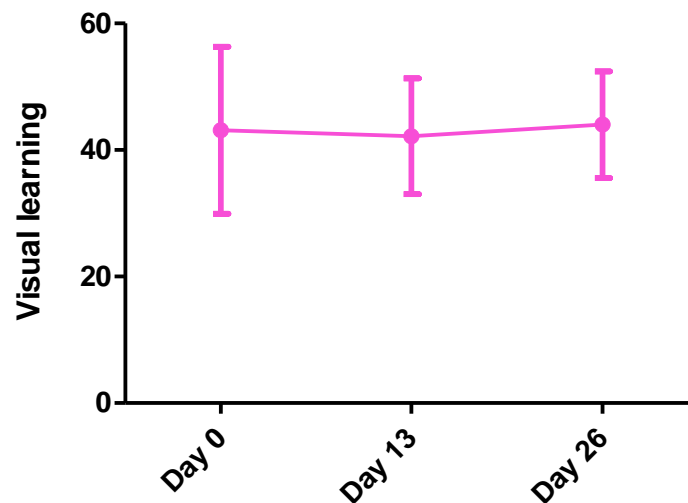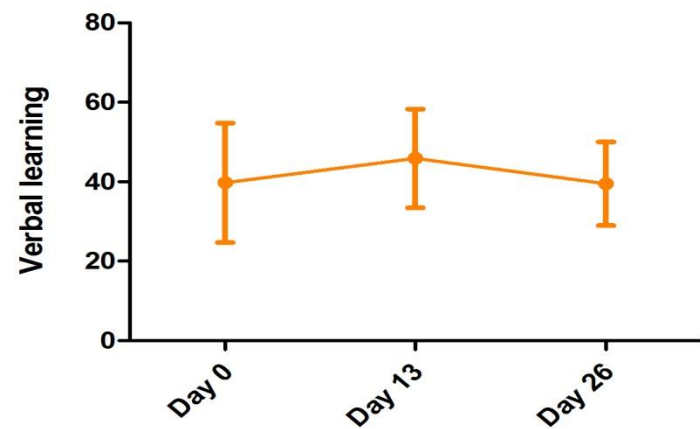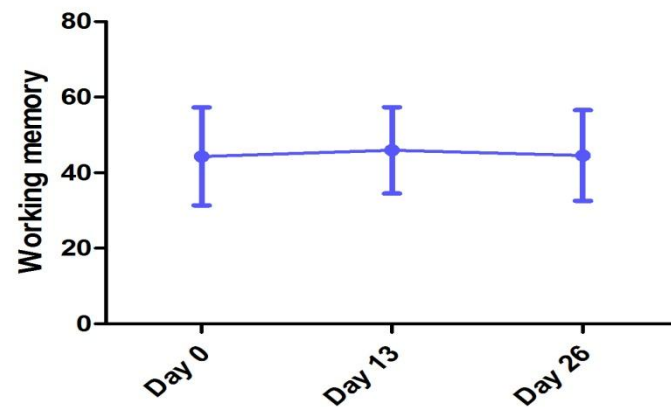

Supplement: Supplemental Information 1 [file peerj-08-10208-s001.pdf]

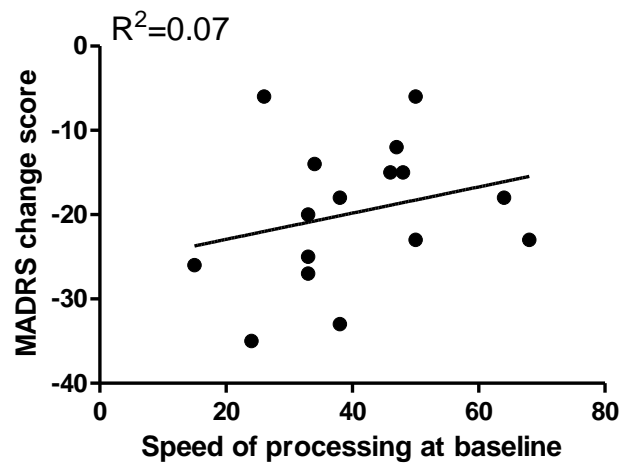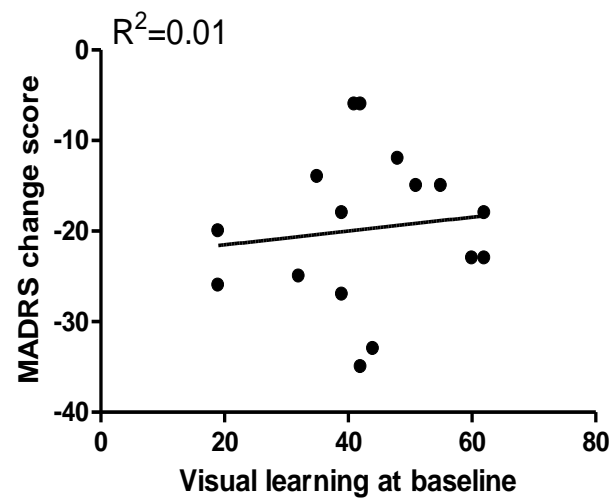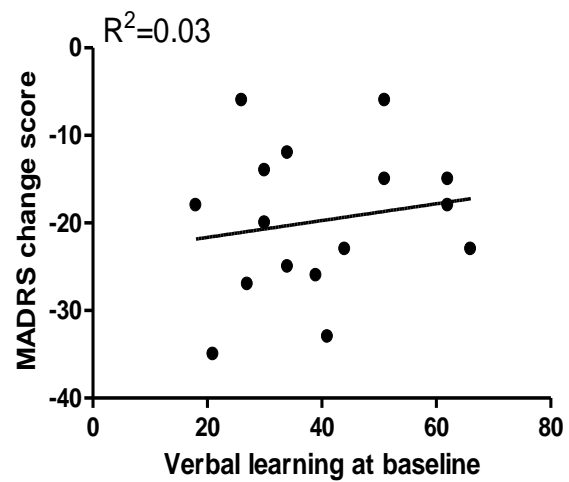

Supplement: Supplemental Information 3 [file peerj-08-10208-s003.pdf]
