# Supplementary material for: Neurocognitive effects of six ketamine infusions and the association with antidepressant effects in treatment-resistant bipolar depression: a preliminary study
Source: PeerJ. 2020 Nov 3;8:e10208. doi: 10.7717/peerj.10208 (PMC7646297; doi:10.7717/peerj.10208)
Supplement: Supplemental Information 2 [file peerj-08-10208-s002.docx]

**Supplemental Table 1. Demographic and clinical characteristics of patients with treatment-resistant bipolar disorder**

| Characteristics | #1 | #2 | #3 | #4 | #5 | #6 | #7 | #8 | #9 | #10 | #11 | #12 | #13 | #14 | #15 | #16 | Total (N, mean±SD) |
| --- | --- | --- | --- | --- | --- | --- | --- | --- | --- | --- | --- | --- | --- | --- | --- | --- | --- |
| Gender, male/female | Female | Male | Male | Male | Female | Male | Female | Male | Male | Male | Female | Male | Female | Male | Male | Male | Female: 5  Male: 11 |
| Married, yes/no | No | No | Yes | No | Yes | Yes | Yes | No | Yes | Yes | Yes | No | Yes | Yes | No | No | Yes: 9  No: 7 |
| Personal monthly income ≥ 4000 yuan/month, yes/no | No | No | No | No | Yes | No | Yes | No | Yes | No | No | No | Yes | No | Yes | No | Yes: 5  No: 11 |
| Employed, yes/no | No | No | No | No | No | No | Yes | No | Yes | No | No | No | Yes | No | Yes | No | Yes: 4  No: 12 |
| Living alone, yes/no | No | No | No | No | No | No | No | Yes | No | No | No | No | No | No | No | Yes | Yes: 2  No: 14 |
| History of psychiatric hospitalization, yes/no | No | No | No | No | No | No | Yes | No | No | Yes | Yes | No | No | Yes | No | Yes | Yes: 5  No: 11 |
| Family history of psychiatric disorders, yes/no | No | Yes | Yes | Yes | Yes | No | Yes | No | No | Yes | No | Yes | No | Yes | Yes | Yes | Yes: 10  No: 6 |
| Major medical condition(s), yes/no | No | No | Yes | No | No | No | No | No | No | No | No | No | Yes | No | No | No | Yes: 2  No: 14 |
| Current smoking, yes/no | No | No | No | Yes | No | Yes | No | No | No | Yes | No | No | No | Yes | No | No | Yes: 4  No: 12 |
| Current drinking, yes/no | No | No | No | No | No | No | No | No | No | No | No | No | No | No | No | No | Yes: 0  No: 16 |
| Age (years) | 23 | 33 | 43 | 23 | 62 | 52 | 47 | 24 | 50 | 40 | 40 | 19 | 37 | 29 | 29 | 54 | 37.8±12.9 |
| Education (years) | 15 | 7 | 5 | 12 | 12 | 13 | 16 | 9 | 15 | 9 | 14 | 12 | 16 | 15 | 12 | 12 | 12.1±3.2 |
| BMI (kg/m^2^) | 21 | 26 | 25 | 28 | 18 | 22 | 22 | 30 | 26 | 28 | 21 | 21 | 22 | 28 | 20 | 21 | 23.7±3.5 |
| Age of onset (years) | 16 | 20 | 37 | 19 | 40 | 20 | 13 | 14 | 30 | 20 | 35 | 14 | 25 | 17 | 19 | 24 | 22.7±8.5 |
| Duration of illness (months) | 84 | 84 | 72 | 37 | 264 | 384 | 408 | 120 | 360 | 360 | 60 | 60 | 144 | 144 | 120 | 360 | 191.3±137.8 |
| Baseline MADRS score | 29 | 20 | 39 | 29 | 28 | 15 | 44 | 20 | 31 | 25 | 29 | 22 | 42 | 31 | 18 | 29 | 28.2±8.3 |
| Speed of processing | 33 | 38 | 24 | 15 | 68 | 47 | 38 | 46 | 50 | 64 | 33 | 48 | 33 | 34 | 50 | 26 | 40.4±14.1 |
| Working memory | 36 | 33 | 38 | 34 | 31 | 69 | 35 | 54 | 60 | 51 | 29 | 53 | 41 | 33 | 66 | 46 | 44.3±13.0 |
| Verbal learning | 27 | 18 | 21 | 39 | 66 | 34 | 41 | 62 | 44 | 62 | 30 | 51 | 34 | 30 | 51 | 26 | 39.8±15.0 |
| Visual learning | 39 | 39 | 42 | 19 | 62 | 48 | 44 | 51 | 60 | 62 | 19 | 55 | 32 | 35 | 41 | 42 | 43.1±13.2 |
| Abbreviations: ADs=Antidepressants; APs=antipsychotics; BMI=body mass index; MADRS= Montgomery-Asberg Depression Rating Scale. | | | | | | | | | | | | | | | | | |
